# Stereotypical bias amplification and reversal in an experimental model of human interaction with generative artificial intelligence

**DOI:** 10.1098/rsos.241472

**Published:** 2025-04-09

**Authors:** Kevin Allan, Jacobo Azcona, Somayajulu Sripada, Georgios Leontidis, Clare A. M. Sutherland, Louise H. Phillips, Douglas Martin

**Affiliations:** ^1^ University of Aberdeen, Aberdeen, UK

**Keywords:** human–AI interaction, stereotypes, large language models, bias in AI, bias amplification, generative AI

## Abstract

Stereotypical biases are readily acquired and expressed by generative artificial intelligence (AI), causing growing societal concern about these systems amplifying existing human bias. This concern rests on reasonable psychological assumptions, but stereotypical bias amplification during human–AI interaction relative to pre-existing baseline levels has not been demonstrated. Here, we use previous psychological work on gendered character traits to capture and control gender stereotypes expressed in character descriptions generated by Open AI’s GPT3.5. In four experiments (*N* = 782) with a first impressions task, we find that unexplained (‘black-box’) character recommendations using stereotypical traits already convey a potent persuasive influence significantly amplifying baseline stereotyping within first impressions. Recommendations that are counter-stereotypical eliminate and effectively reverse human baseline bias, but these stereotype-challenging influences propagate less well than reinforcing influences from stereotypical recommendations. Critically, the bias amplification and reversal phenomena occur when GPT3.5 elaborates on the core stereotypical content, although GPT3.5’s explanations propagate counter-stereotypical influence more effectively and persuasively than black-box recommendations. Our findings strongly imply that without robust safeguards, generative AI will amplify existing bias. But with safeguards, existing bias can be eliminated and even reversed. Our novel approach safely allows such effects to be studied in various contexts where gender and other bias-inducing social stereotypes operate.

## 1. Introduction

In the future, artificial intelligence (AI) systems might make societies fairer by providing consistently objective advice in situations where social stereotypes have historically led to discrimination and prejudice [1–4]. To reach that happy state, behavioural scientists must first deal with specific and widespread concerns about the psychological impacts of human–AI interaction [5,6]. A key driver of concern is that deep learning allows AI systems to acquire and express biases in training data [5–7] that reflect social stereotypes, as exemplified by racially biased recidivism prediction [8,9], or gender-biased hiring recommendations [10–13]. Finding machine learning solutions that remove or mitigate such bias is highly challenging [14]. But we should recognize that even if machine learning solutions are gradually developed, it is simply naive to believe that *all* AI systems will include such safeguards [5,15]. Thus, we can reasonably expect that stereotypically biased systems will continue to influence human decision-making, and it is therefore crucial that we understand and mitigate their effect.

The need for such understanding has never been so timely. Rapid developments in generative AI are embedding the technology ever deeper into society [16], attracting hundreds of millions of new users globally [17]. In this context, it is hugely concerning that evidence of systematic stereotypical bias in the output of generative AI is accumulating [18–24], when attempts are made to harness systems to perform specific tasks involving summarizing or interpreting existing text to extract new insights from it or to provide different perspectives upon it. Evidence of biases discovered so far involves various stereotypes relating to disability, regional dialects, race and historically stable binary conceptions of masculine and feminine character, as well as some surprisingly subtle forms of bias reflecting relatively recent shifts in feminine stereotypes [25]. These findings raise an unfortunately plausible alternative future scenario. AI systems may persuasively reinforce human stereotypes and thereby amplify their influence on decision-making, leading to less fair future societies. While this seems an entirely reasonable prediction, it is based on a hypothetical phenomenon—amplification of human stereotypical bias by similarly biased generative AI—that has not yet been established.

What is well-established is the human tendency to seek out information confirming our own views [26–31] and to conform to others who express such views [29,32–43]. When these tendencies are supported or exploited by biased technology, we should not be surprised to find bias amplification phenomena [44]. For sound ethical reasons, however, we cannot study bias amplification in human–AI interaction by releasing biased systems into consequential decision-making contexts. But there are a few known cases where this happens unintentionally, and biased systems temporarily operate without safeguards until withdrawn [5,11,45,46]. Assessing the impact of these systems is highly complex [47,48] and overlaid by issues of commercial sensitivity and reputational loss. These factors make it difficult to quantify whether the biased systems were persuasive, and to whom, or account for pre-existing stereotypical bias in humans who interacted with the systems. While these ‘natural experiments’ offer startling insights into the societal impact of biased AI and demonstrate how urgent it is to confirm the reality of bias amplification in human–AI interaction, their very existence exposes a continued failure to understand and rectify the problem.

Perhaps the only way to develop this understanding, and ultimately reach a fairer society with AI, is to establish and study relevant phenomena in human–AI interaction under experimentally controlled conditions. In so doing, our approach here explicitly departs from the assumption that technocentric solutions—focused on refining model architectures and training data—will be sufficient to tackle biases in large language models (LLMs). We do not expect that bias-free performance will be a reliable and general feature of such systems anytime soon, so we focus on understanding how biased systems influence human judgements. By studying human interaction with biased systems, our aim is to identify strategies to reduce bias propagation and also to enhance the persuasiveness of potentially neutral or debiased systems, particularly when their debiased outputs are ‘misaligned’ with prevailing cultural stereotypes.

In the four experiments we report here, we focus on bias propagating from generative AI that provides specific text-based outputs to support human decision-making. This application of generative AI, and the resulting human–AI interactions that it produces, has become the predominate use for LLMs, including leading models such as Open AI’s GPT series. Although LLMs are now famous for their ability to hold human-like conversations, perhaps counter-intuitively they are not predominately being used to perform specific tasks that involve free conversations with humans [49,50]. Instead, LLMs are predominately being used to provide specific, tailored outputs, using tightly constrained prompts to control and stabilize their behaviour (i.e. their outputs). This allows them to be benchmarked within particular tasks, revealing their various systematic properties, including a propensity towards stereotypical biases [18–25]. A key feature of our experimental approach, going beyond existing work on human–AI interaction [51], is that we have developed a prompt-based method of controlling the expression of gender stereotypes in the natural language output by an LLM as it performs a specific task. We describe the psychological basis of the method next. Following that we describe our initial validation and replication experiments, which demonstrate that we can significantly amplify, or reverse, human participants’ baseline gender stereotypical bias when they interact with an experimentally simulated recommender system that we can configure to reinforce or challenge gender stereotypes.

**Figure 1.**
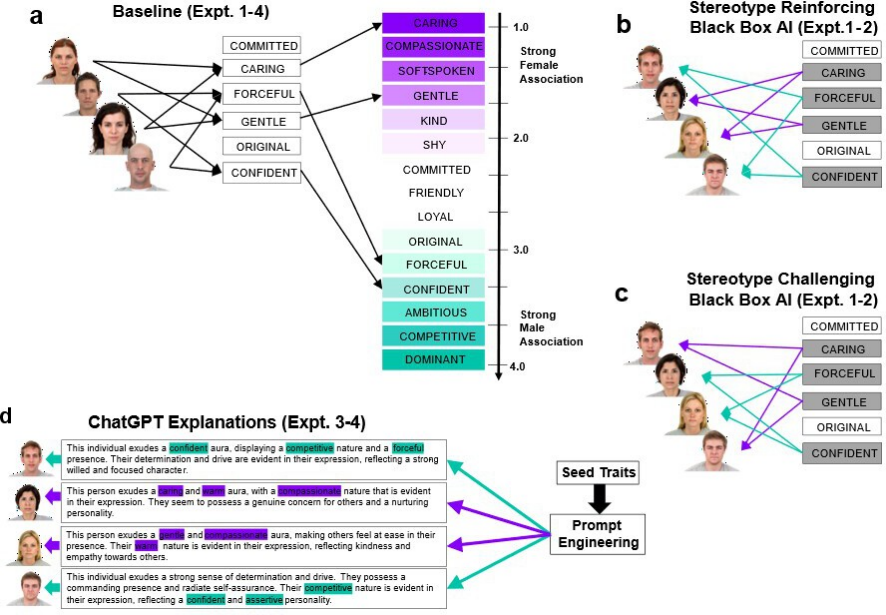
Experimentally simulating human interactions with stereotypically biased AI. Historically, particular character traits have been associated with binary conceptions of the character of men versus women. We used ratings of how strongly these traits are associated with each gender to precisely control the stereotype-reinforcing, neutralizing or challenging bias of a simulated recommender system helping human participants to judge people’s character from their faces. (a) In all four experiments, participants first viewed facial images of unfamiliar men and women, selecting traits from the list that fit their first impression of each individual. Every trait’s pre-rated 55 gender stereotype association (GSA) score captures the strength of its association with male or female stereotypes, with traits strongly linked to women scoring low and traits strongly linked to men scoring high. Participants’ baseline stereotypical bias is quantified via divergence in the mean GSA score of all the chosen traits for women versus men. Here we illustrate, via the directed black arrows, a participant with a stereotypically biased baseline response pattern, whose first impressions of men consistently involve traits strongly linked to males and vice versa for women, with each chosen trait mapped back onto the GSA scale. (b) Augmented phase, following the baseline phase, where new target faces are shown, each with a pair of highlighted traits (experiments 1–2) recommended in a ‘black-box’ manner (i.e. without explanation). Participants are free to select any pair of traits, whether recommended or not. The directed arrows illustrate a stereotypically biased recommendation pattern that consistently reinforces female (purple arrows) and male (green arrows) gender stereotypes. Relative to baseline, changes (e.g. amplification) in stereotypical bias are quantified by the mean GSA score of traits chosen for men versus women during the human–AI interaction. (c) Complete control over the simulated bias pattern expressed in recommendations is easily achieved as illustrated by this counter-stereotypical recommendation pattern, which consistently challenges gender stereotypes by always recommending male-typical traits (green arrows) for women and female-typical traits (purple arrows) for men. (d) Prompt engineering in experiments 3 and 4 was used to seed a generative AI (Open AI’s GPT3.5) with character traits to produce descriptions of people in natural language with precise control over how stereotypical (of men or women) they were. Each character description was seeded with three traits, and the examples shown here all use strongly male-typical (green arrows/highlights) or female-typical (purple arrows/highlights) traits to create a consistent stereotype-reinforcing influence upon participants’ first impressions and corresponding responses. Participants were shown a GPT3.5 description for each target, to aid the formation of their first impression.

Psychological studies have identified stable features of gender stereotypes that persist despite seismic societal changes since the end of the Second World War. Linked to women’s rights and changing roles at home and in the workplace, stereotypes of female characters have shifted towards increased agency and competence [52,53]. But women are still strongly associated with a range of traits signalling communion and warmth, while men are still, more strongly than women, associated with traits signalling agency and competence [52–55]. These persistent associative patterns existed before the Internet and continue to the present day, which accounts for the particular vulnerability of deep learning systems to biases in training data that derive from these stable features [56,57]. While the treatment of gender diversity in ethical AI frameworks [14] extends beyond the binary conception that comes with such stereotypes, it is appropriate that we focus on them here because their historical stability and resulting penetration into training data are precisely what ethical AI frameworks are trying to deal with. Understanding the propagation of this, so to speak, ‘inherited’ bias, is, therefore, a necessary step. So, here we exploit psychological work on the stable features of gender stereotypes to deliberately engineer, completely remove or even reverse gendered biases within an experimentally simulated recommender system, providing the precise control needed to establish and study bias propagation within human–AI interaction (figure 1).

Our choice of experimental task reflects the fact that stereotypes have the most influence on the perception of unfamiliar individuals about whom we have little or no other knowledge. The effect of gender stereotypical bias in AI is therefore most potent and most difficult to counteract when it influences inferences about the underlying character of strangers. We use the long-established [28] experimental analogue for such situations, a first impressions task, which participants perform initially on their own, and then augmented with advice from AI recommender systems imbued with simulated bias patterns (figure 1a). Participants view facial images of unfamiliar men and women and choose character traits that fit their first impression of each person from a short list of selectable trait options. These traits map onto the stable features of male and female stereotypes [55], allowing a measure of stereotypical influences on first impressions. Participants’ baseline stereotypical bias is thus quantified by their tendency to select traits strongly associated with men for first impressions about men and vice versa for women. This baseline sets the standard against which we can measure changes (e.g. amplification) of bias when the task is performed with advice from different types of recommender systems whose bias is under our control. Because our focus here is on the propagation of gender stereotypes, it is incumbent upon us to control other potentially intersecting forms of stereotypical bias that could moderate gender stereotypes, and in particular, racial or age-related stereotypes. For this reason, we use facial images of young, White men and women throughout the experiments reported here, which provides a basis for future studies that could examine intersectionality effects in human interaction with biased AI [58,59].

In experiments 1 and 2, we begin by establishing whether stereotypically biased character recommendations using single traits significantly amplify the influence of gender stereotypes on first impressions beyond their baseline expression. Four different between-groups manipulations were implemented to simulate a recommender system for this task with different inherent biases. One group received two stereotype-reinforcing AI recommendations on every augmented trial—i.e. for male targets only strongly male-linked traits were recommended, and for female targets, only strongly female-linked traits were recommended (figure 1b). In this ‘fully reinforcing’ condition, the simulated AI was maximally stereotypically biased. For the second group, the bias was entirely reversed to create a counter-stereotypical influence that always challenged stereotypes. In this ‘fully challenging’ condition, the two recommended traits for each female target were strongly male-linked and vice versa for male targets (figure 1c). In the remaining two groups, a neutral trait was recommended alongside either a male-linked trait for male targets or a female-linked trait for female targets (partially reinforcing), or vice versa (partially challenging).

## 2. Methods (experiments 1 and 2)

### 2.1. Participants

All experiments reported here were conducted online and approved by the Aberdeen University School of Psychology Ethics Committee. Each participant gave their informed consent and was remunerated with either 3 or 3.5 US dollars for a 20 min session, depending on the recruitment platforms’ expected equivalent hourly compensation rate of 9 or 10 US dollars. Recruitment was restricted to participants validated for age and gender by the host platform, aged 18+ years and either native English speaking or highly fluent. The only other inclusion criterion, on the country of residence, is described where applicable per the experiment below.

Experiment 1 recruitment used the online participant panel ‘Testable Minds’ [60]. We required four participant groups, one for each simulated bias condition that was manipulated between group. Our target in each of the four groups was at least an *n* = 48, based on effective sample sizes used in our prior work on human–AI interaction and social influences driving conformity to other humans [39,41,61]. In total for experiment 1, we recruited 205 people, excluding one participant because they self-reported that they were not fluent in English and one because of the outlying length (greater than 50 min) of their session duration. In the remaining 203 participants, the mean age was 29.1 years (18–74), 102 were male (mean age 28.7, 18–71 years), 98 were female (mean age 29.6, 18–74), with three self-reported as other. Casting the net as widely as possible in terms of recruitment, we did not restrict nationality, ethnicity or country of residence but (given the task) recruited solely on the basis of English language competency. Our sample was highly diverse, with 68% of participants reporting nationality from non-English-speaking countries and 32% from either the UK, the USA, Canada, Australia or Ireland. On ethnicity, 74.9% were non-White. In total, 51.7% of participants reported English as their native language, while the rest self-reported as fluent English speakers.

In experiment 2, which sought to replicate key findings from experiment 1, we used a different online participant panel, Prolific [62], but the same experiment generator platform (Testable). Our recruitment target was to double the group *N* to at least 96, focusing on the two fully biased conditions, i.e. ‘fully reinforcing’ and ‘fully challenging’. In total, we recruited 193 participants, of which two were excluded using the same session duration cut-off (i.e. 50 min). We recruited participants aged 18+, in two different age groupings within each simulated bias condition—one younger and one older, which allowed us to examine any age-related changes in the pattern of effects. No age-related effects were present, however, and in all analyses reported here, the two age sub-groups within each bias condition are combined into one group. In the full sample of 191 participants, we had 95 male, 92 female, 4 self-reported as other and an overall mean age of 52 years (range 19−94 years; see footnote^1^). For nationality, 87.4% gave either Canada, Ireland, the UK or the USA. On ethnicity, 88.5% were White with 96.9% reporting that English was their native language.

### 2.2. Procedure

Following consent and initial instructions, participants provided demographic information on their age, gender, native language/English fluency, nationality, ethnicity and sexual preference. They then performed a first impressions task that had two phases separated by a brief minute’s rest. In the first, baseline, phase, 24 experimental trials were given, 12 showing a male face and 12 a female face (in a differently randomized order for each participant). Each baseline trial began with the onscreen display of a face under which six individual trait response options were shown, and participants were instructed to use the mouse/trackpad on their laptop/desktop PC to respond by selecting two traits that best fitted their first impression of the individual whose facial image was shown on that trial. This task was entirely self-paced with no response-time restriction. As soon as the required number of traits had been selected, a ‘CONFIRM’ button appeared, and when participants clicked on that, the screen was blanked and then the next trial began immediately. Prior to the 24 baseline trials, a set of four practice trials (two containing male faces and two containing female faces) was given to familiarize participants with the procedure.

After a brief self-paced rest interval, the augmented phase instructions were shown. Common to all experiments, participants were instructed that their first impression task would now be aided by recommendations provided by an AI. This mild instructional deception was used to conceal the fact that the recommendations were actually constructed prior to the experiment, as described in detail in §2.3. The format of these recommendations varied across experiments (see experiment-specific details). Every augmented phase contained 24 experimental trials, in which 12 male and 12 female facial images were shown (involving an entirely different set of images to those shown at baseline), which were again preceded by four practice trials involving two male and two female facial images. The procedure for these augmented trials was identical to the baseline in every respect except for the addition of the AI’s recommendations. Participants were instructed that they were free to use the recommendations, or not, as they wished. Once the augmented phase was complete, a set of exit questions in Likert or free text form was administered to gather qualitative data on the participant’s views about their experience of the experiment, their views on the AI and the recommendations and also their year of birth. These data are not included for analysis here. Testable’s data-saving parameter was set to only save complete and not partial datasets.

### 2.3. Materials

All four experiments were conducted online via Testable, advertised to potential participants with a brief description of the experimental purpose to investigate human–AI interaction. Participation required a laptop or desktop PC and not any other form of device. On accepting recruitment, initially, each participant filled in an online consent form, and if they agreed to consent, then they were shown instruction screens. Each participant in each experiment was told that the purpose was to study how AI can aid human decision-making (see experiment-specific details below for per-experiment instructions).

Each experiment used the same first impressions task, involving a set of male and female facial images and a set of 48 character description traits. The 48 traits had previously been rated in terms of the strength of their association with male and female stereotypes [55]. On each trial of the first impressions task, participants were shown either a man’s or a woman’s face and a set of response traits they should use to indicate their first impression of that person’s character (see figure 1 and experiment-specific methods below for details on the traits shown in each experiment). The facial images were taken from the Chicago Face Database (CFD) [63]. In all experiments, the facial images depicted young White adults, sampled from the CFD.

The set of 48 character traits originates from prior studies in our laboratory [55] where each trait had been rated in terms of how strongly it was associated with either male or female stereotypes using a single scale 5-point Likert scale. The low anchor point is ‘highly associated with female stereotype’, the high anchor point is ‘highly associated with male stereotype’ and the midpoint is ‘associated with both or neither stereotype’. The mean gender stereotype association (GSA) score provides a metric for the strength of stereotypical association for each individual trait, which can be used to control and manipulate stereotypical bias in subsets of traits and induce such biases within human decision-making [55].

Humans readily and quite naturally use faces to judge character attributes [64], and here we capitalized on this by simulating different patterns of stereotypical bias within an ‘AI recommendation system’ that participants were led to believe provided analyses of facial images of men and women to infer and summarize their character. Participants were instructed that the AI’s role was to provide summary recommendations on character in the form of single-word traits—i.e. our set of 48 stereotype-linked character traits—to aid the formation of first impressions on the experimental task. Participants were not provided with any further information at all about the underlying system—e.g. about the training, internal workings, accuracy, limitations or applications of the system.

In reality, the recommendations were constructed prior to the experiment using the following method. In experiments 1 and 2, six selectable traits were shown on each trial (figure 1b). Two of these were drawn from the GSA scale top end (strongly male stereotyped), two from the middle (neutral) and two from the bottom end (strongly female stereotyped). To fully utilize all 48 traits, we exploited the combinatorial fact that 28 unique pairs of items can be drawn from a parent set of eight items. Thus, we split the top 16, middle 16 and lower 16 on the scale at random into an A and a B set each of eight items. We then formed the 28 unique pairings available from each set of eight, and then we randomly combined pairs of male-linked, neutral and female-linked traits to produce 28 sets of six traits from the A lists and 28 sets of six traits from the B lists. Half of the participants then saw the A-list 28 × 6 trait sets at baseline and the B-list 28 × 6 trait sets in the augmented phase, and vice versa for the remaining participants.

A similar counterbalancing was used to remove any accidental correspondence between male and female facial images and the six trait sets. The original set of 56 (28 men and 28 women) facial images was split into an A and a B set each containing 14 men and 14 women. The A and the B facial image sets were then used equally often in the baseline and augmented phases (including the four practice trials that preceded each phase), with an additional counterbalancing to ensure that each set of six traits was shown equally often with a facial image of a man or a woman. These measures resulted in eight different counterbalancing conditions underlying the repeated measures factors within each bias condition; that is, phase (baseline/augmented) and target gender (man/woman). At the beginning of each online experimental session, each participant was randomly assigned to one of these counterbalancing conditions by Testable with equal probability.

In experiment 1, four different bias patterns were simulated to manipulate whether male and female stereotypes were being reinforced or challenged to different degrees. In the ‘fully reinforcing’ condition, recommendations always used character traits strongly associated with the gender of the person depicted in each facial image. For images of women, recommendations always used the pair of traits within the six trait response set on the trial that came from the low, strongly female stereotypical end of the GSA scale. For images of men, recommendations always involved the pair of traits on the trial that came from the high, strongly male stereotypical part of the GSA scale. By reversing the mapping between facial gender and the type of traits being recommended, i.e. recommending female-typical traits for every target man and recommending male-typical traits for every target woman, the bias pattern was completely reversed becoming counter-stereotypical in the ‘fully challenging’ condition.

In the two partial bias conditions, i.e. ‘partially reinforcing’ and ‘partially challenging’, one recommended trait was a middle-scoring, neutral item and the other was either male-/female-typical for images of men/women (‘partially reinforcing’) or the reverse (‘partially challenging’). In experiment 2, the replication study, we utilized the two extremes, i.e. ‘fully challenging’ and ‘fully reinforcing’ bias conditions. The recommendations were provided, in both experiments, by highlighting two of the six onscreen response traits, reflecting the AI’s summary advice about the character of the individual shown in the facial image (figure 1b). The recommended traits were highlighted continuously from the beginning of the trial until the participant clicked on the ‘CONFIRM’ button to enter their selections and initiate the next trial. The replication study (experiment 2) focused on the two fully biased conditions and did not utilize the two partially biased conditions.

## 3. Results

### 3.1. Experiment 1

**Figure 2.**
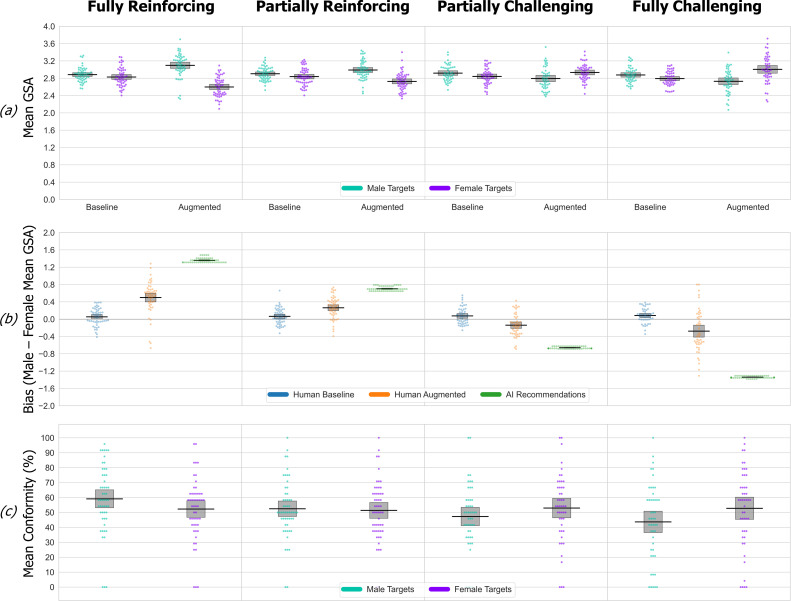
Experiment 1 data. Here and in subsequent figures the data are organized into columns, one for each simulated AI bias condition. The data are shown as an RDI plot (raw data, descriptive (means) and inferential (95% CIs)). (*a*) Mean GSA scores of traits participants selected for male versus female targets at baseline and augmented phases in each of the four simulated AI bias conditions. Higher/lower GSA scores indicate selection of traits with stronger association with male/female stereotypes. Error bars show the 95% CI (confidence interval) in all panels and in every subsequent figure. (*b*) For each simulated AI bias condition, a direct comparison of participants’ bias (male–female GSA score difference) at baseline and augmented phases, along with the total ‘available’ simulated bias (i.e. the difference in mean GSA score of all recommended traits for male–female targets). (*c*) The mean percentage conformity rates for recommended traits, i.e. how often, in each simulated bias condition, participants actually selected the traits that were recommended to them for male versus female targets.

The dataset for this and the subsequent experiments are available online [65]. An initial mixed-design ANOVA of the mean GSA score for selected traits used between-group factors of simulated AI bias and within-participant factors of target gender (male versus female) and phase (baseline versus augmented). The resulting three-way interaction (*F*_3, 199_ = 46.15, *p* < 0.001, partial *η*² = 0.41) reflects a consistent baseline bias present in all four groups that qualitatively changes in each augmented phase as decisions became influenced by the different simulated AI bias patterns. The patterns at baseline and in the augmented phases in mean GSA scores are shown in figure 2*a*. The first key point is that at baseline, each participant group had a significant bias towards utilizing stereotypical traits, i.e. higher mean male versus female GSA scores. This difference (i.e. the baseline bias) in the mean male versus female GSA score was statistically significant within each group (simple effects pairwise comparisons, maximum *p* = 0.035, Bonferroni-corrected) and did not itself differ between groups (*t*s_99_ ≤ |0.81|, *p*s ≥ 0.42, Cohen’s *d* = 0.16). The presence of a significant baseline bias replicating in all four independent groups highlights our task’s sensitivity and ability to detect a pre-existing stereotypical baseline bias across diverse adult samples.

The second key point is that compared with the mean male and female GSA scores at baseline, there was a significant shift in the corresponding augmented phase mean male and female GSA scores in every simulated bias condition (simple effects pairwise comparisons, maximum *p* = 0.005, Bonferroni-corrected). The third key point is the differential impact of the simulated AI-bias conditions in the augmented relative to baseline phases. This is shown in figure 2*b*, which highlights the amplification and reversal of the baseline pattern in each group in the reinforcing versus challenging conditions. The participants’ augmented phase bias was significantly different in each pairwise comparison across the AI-bias conditions (*t*s_105_ ≥ |3.83|, *p*s ≤ 0.001, Cohen’s *d* ≥ 0.74) except for the fully versus partially challenging conditions where the effect is weaker (*t*_94_ = 1.72, *p* = 0.09 (two-sided), Cohen’s *d* = 0.35).

How effectively does the simulated bias within the recommendations propagate into participant’s decisions? We can quantify this by measuring participant’s augmented phase bias as a proportion of the ‘available’ bias within the recommendations. This measure was significantly higher (*t*s_100_ ≥ |2.17|, *p*s ≤ 0.032, Cohen’s *d* ≥ 0.43) in both of the stereotype-reinforcing conditions (fully = 0.37 (s.d. = 0.28), partial = 0.38 (s.d. = 0.36)) compared with the challenging conditions (fully = 0.21 (s.d. = 0.36), partial = 0.21 (s.d. = 0.40)). The influence exerted by the recommendations can also be examined via the conformity data, measuring how frequently participants agreed with target recommendations (figure 2*c*). For female targets, conformity remains essentially constant across all four simulated AI-bias conditions. For male targets, conformity to stereotype-challenging recommendations is much reduced compared with stereotype-reinforcing recommendations. Supporting this interpretation, target gender interacted with simulated AI-bias condition (*F*_1,199_ = 9.46, *p* < 0.001, partial *η*² = 0.16), because AI bias has no effect on conformity to recommendations about female targets (simple effects pairwise comparison, all *p*s = 1, Bonferroni-corrected), whereas conformity to recommendations about male targets was significantly higher in the fully reinforcing versus both challenging conditions (*p*s ≤ 0.05, Bonferroni-corrected).

### 3.2. Experiment 2 (replication results)

**Figure 3.**
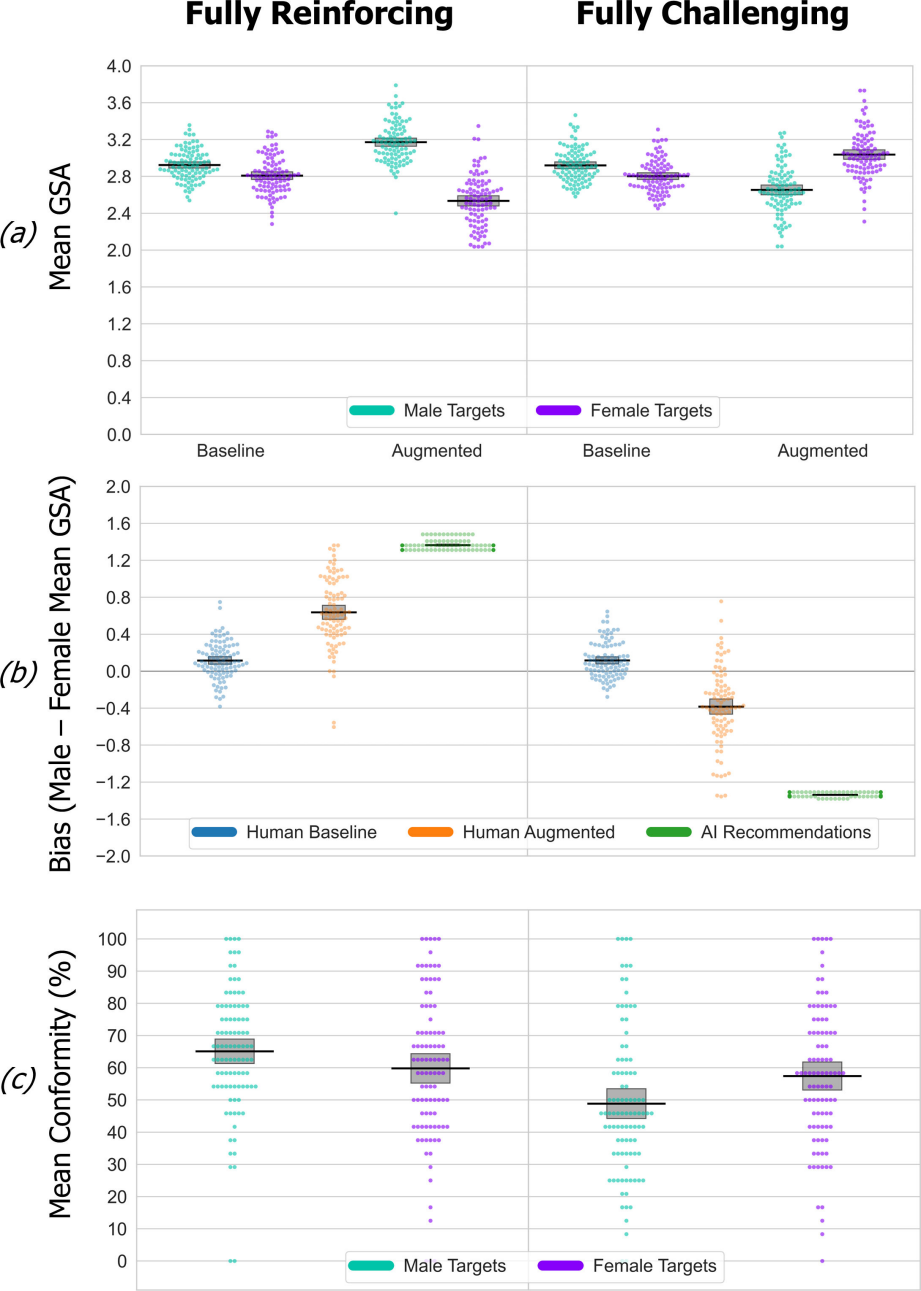
Experiment 2 (replication study) data. See figure 2 legend for details. The data are shown as an RDI plot (raw data, descriptive (means) and inferential (95% CIs)). The replication study was entirely successful in confirming the findings of the original experiment 1. (*a*) Fully stereotype-reinforcing recommendations strongly associated with male/female stereotypes significantly increased participant’s selection of stereotyped traits in the augmented phase, correspondingly shifting their mean GSA scores upwards for male and downwards for female targets, compared with baseline. Fully stereotype-challenging recommendations significantly reversed the augmented phase mean GSA scores, i.e. they reversed the direction of participant’s baseline bias. (*b*) Participant’s baseline bias is statistically significant within each group and does not differ between groups. Participants’ augmented phase bias is significantly different in each simulated AI-bias condition. (*c*) Stereotype-reinforcing versus challenging simulated AI bias has no effect on conformity to recommendations about female targets, but does for male targets (conformity to recommendations is significantly higher in the fully reinforcing versus challenging condition).

The findings from experiment 1 are novel, and so in experiment 2, we provide a scaled-up replication of the bias amplification and reversal effects in the fully challenging and reinforcing conditions. Using a different online recruitment panel (Prolific), the recruitment target *N* in each group was doubled from 48 to 96. Experiment 2 findings are shown in figure 3. Mixed-design ANOVA of the GSA data, using between-group factors of simulated AI-bias (fully reinforcing versus fully challenging) and within-participant factors of target gender (male versus female) and phase (baseline versus augmented) gave a significant three-way interaction and increased effect size reflecting the focused contrast between the fully reinforcing versus challenging AI-bias conditions (*F*_1,189_ = 300.79, *p* < 0.001, partial *η*² = 0.61). The interaction reflects a transition from consistent baseline patterns into radically different augmented phase patterns as the participant’s decision-making is pulled towards the opposing forms of bias embedded in recommendations.

Figure 3*a* shows the consistent pattern of baseline bias, and significant shifts from baseline induced by the AI bias patterns that exactly replicate experiment 1’s findings (compare figure 2*a* with figure 3*a*). Participant’s baseline bias is statistically significant within each group (simple effects pairwise comparison, all *p*s < 0.001, Bonferroni-corrected) and does not differ between groups (*t*s_189_ ≤ |0.04|, *p*s ≥ 0.97, Cohen’s *d* ≤ 0.006). Stereotype-reinforcing recommendations (figure 3*a*, left) significantly increased and decreased participant’s augmented phase GSA scores for male and female targets, respectively, compared with their baseline scores (simple effects comparison, all *p*s < 0.001, Bonferroni-corrected). While stereotype-challenging recommendations (figure 3*a*, right) significantly reverse the direction of participant’s baseline bias (simple effects comparison, all *p*s < 0.001, Bonferroni-corrected). Hence, the participants’ augmented phase bias (shown directly in figure 3*c*) is significantly different in each simulated AI-bias condition (*t*_189_ = 17.87, *p* < 0.001, Cohen’s *d* = 2.59).

The significantly increased propagation of stereotype-reinforcing versus challenging influence also replicates here, taking participant’s augmented bias as a proportion of the available simulated bias in recommendations (0.46 (s.d. = 0.28) versus 0.29 (s.d. = 0.31), *t*_189_ = 4.32, *p* < 0.001, Cohen’s *d* = 0.63). Experiment 1’s conformity data pattern also replicates here, where attempting to challenge male but not female stereotypes has reduced conformity to recommendations (figure 3*c*). Conformity rates were analysed via ANOVA, using target gender and simulated AI-bias condition as factors, which significantly interacted (*F*_1,189_ = 20.64, *p* < 0.001, partial *η*² = 0.1). The interaction occurs because the AI-bias condition has no effect on conformity to recommendations about female targets (simple effects pairwise comparison, *p* = 0.46, Bonferroni-corrected). Whereas, for male targets, conformity to recommendations is significantly higher in the fully reinforcing versus challenging condition (*p* < 0.001, Bonferroni-corrected). In sum, experiment 2 precisely replicates experiment 1’s baseline bias, bias amplification, bias reversal and conformity findings within the fully reinforcing versus challenging simulated bias conditions.

### 3.3. Discussion (Experiments 1 and 2)

Experiment 1’s primary contribution is to establish stereotypical bias amplification and reversal in an experimental model of human–AI interaction. Stereotypical bias amplification beyond a relevant human baseline level has not previously been demonstrated in human–AI interaction, although it is clearly a prediction on which many different strands of work converge from human psychology [27–31,38,44,55,61], studies of algorithmic bias [5,10,18,19,21–23,56] and augmented decision-making [9,47,48,51,66]. Here we provide the first direct empirical evidence of the core phenomenon—bias amplification—driving public and expert concern about human–AI interaction. A further notable finding is the observed reversal of baseline bias in the stereotype-challenging conditions, which points (rather more encouragingly) to the likely effectiveness of debiasing measures in counteracting pre-existing human stereotypical bias.

Experiments 1 and 2 establish a robust way to safely introduce and manipulate highly persuasive expressions of gender stereotypical content within human–AI interaction, demonstrating its significant influence relative to pre-existing, baseline levels of stereotyping. Our approach not only shows that a stereotype-reinforcing AI bias significantly amplifies a pre-existing baseline human bias, but it also captures the unfortunately heightened propagation of the stereotype-reinforcing bias compared with the stereotype-challenging bias of equal (but opposite) magnitude, in already biased groups of human participants. These data again link the reduced influence of counter-stereotypical messaging to more robust male character stereotypes, which fits well with gender-equality work that has focused on combating female but not male stereotypes. Huge efforts in fair-AI research are currently devoted to the removal of bias from AI systems, and our results in the stereotype-challenging conditions indicate that debiased systems can effectively reverse baseline human bias, albeit at a cost to their persuasiveness.

It is worth pointing out that relative resistance to stereotype challenge for male targets is not a general reluctance to apply stereotypically female traits to strangers, because no corresponding reluctance occurs when applying such traits to female targets. What we are instead observing is greater *attachment* to male stereotypes, expressed as increased endorsement of reinforcing recommendations and reduced endorsement of challenging recommendations. It seems reasonable to suggest that the relative robustness of male versus female stereotypes to challenge could reflect the gender-equality efforts cited in the introduction that have, for decades, directly challenged female but not male stereotypes [52,53]. Furthermore, the relative ease with which challenging recommendations are accepted for female targets conforms very well with the ‘surprising’ gender biases recently documented by Fulgu & Capraro in the output of GPT3.5 and GPT4.0 [25]. Fulgu & Capraro observed that these models were statistically more likely to assign women traditionally masculine roles than assign traditionally feminine roles to men, in a task which prompted the models to imagine who authored brief pieces of text (such as ‘My favorit color is pink!’).

## 4. Introduction (experiments 3 and 4)

Our next step is to determine whether we can control and manipulate the expression of gender stereotypes by generative AI and experimentally establish similar bias amplification and reversal phenomena. For this aim, we utilized Open AI’s GPT3.5 model (GPT3.5 turbo version, freely available). GPT3.5 has been trained using reinforcement learning with human feedback, supplemented by guard-rails within its ChatGPT version, to immediately terminate conversations categorized as unethical, including the expression of gender stereotypes and most particularly their toxic expression. Systematic, non-toxic expressions of various stereotypes, however, are not particularly difficult to elicit from GPT models, including disability stereotypes [18], racial stereotypes [19] and gender stereotypes [20,21,24,25], using simple prompts that guide it to perform specific tasks.

Here we utilized a prompt engineering approach that involved seeding GPT3.5 with the character traits used in experiments 1 and 2 to produce non-toxic but nevertheless extremely stereotypical descriptions of men and women (see figure 1d and §2), as well as neutral descriptions using traits without any strong linkage to either male or female stereotypes. Via this process, we created a large set of character descriptions in natural language that attempt to express male and female character stereotypes with high fidelity.

The fidelity of GPT3.5’s expression of gender stereotypes from the trait seeds is very important. It is possible that gendered character stereotypes might be weakened or distorted when GPT3.5 elaborates the seed traits into character descriptions. It is also quite possible that the bias patterns we intend to create might be strengthened, perhaps even caricatured, by any inherent bias within GPT3.5 itself. To maintain experimental control, we had to constrain such possibilities, and so we insisted within the prompt engineering that the trait seeds had to be used verbatim. Under that constraint, it becomes possible to compare the influence of GPT3.5’s character descriptions with the ‘pure’ influence of the trait recommendations used in experiments 1 and 2. This leads us to predict that we should see essentially the same bias amplification and reversal patterns here in experiment 3.

### 4.1. Methods (experiments 3 and 4)

#### 4.1.1. Participants

In experiment 3, we returned to using the Testable Minds online participant panel (with participation in experiments 1 or 2 as an exclusion criterion). Aside from experiment 1 and 2 results, we had no prior guidance on effect or sample size for conformity to natural language recommendations, so we increased the target group *N* to at least 64 (as compared with *n* = 48 in experiments 1 and 2). This was a precautionary measure to offset any decreases in statistical power if effect sizes were potentially reduced for bias propagation via GPT’s natural language recommendations. We required three participant groups, one for a ‘fully reinforcing’ bias condition, one for a ‘fully challenging’ bias condition and one for a ‘neutral’ (no bias) condition. For experiment 3, we obtained a sample of 196, of which four were excluded due to extreme outlying session duration (greater than 50 min). Of the remaining 192 participants, 103 were male (mean age 35.9 years, 18–66), 86 were female (mean age 39 years, 19–68) and three were others. Recruitment was focused on countries where English is the official state language, and 90.7% gave nationality as either the UK, the USA, Canada, Ireland or Australia, and 70.8% of the sample’s self-reported ethnicity was White.

In experiment 4, we obtained a new sample of 199 participants, excluding three due to outlying session length (greater than 50 min). Of the remaining 196 participants, 100 were male (mean age = 35.7 years, 18–70), 92 were female (mean age = 39.5 years, 21–80) and four were other). We again focused recruitment on officially English-speaking countries, resulting in 92.4% giving nationality as either the UK, the USA, Canada, Ireland or Australia, and 76.5% of the sample self-reported as of White ethnicity.

#### 4.1.2. Procedure

All procedural details in the baseline and augmented phases were exactly as per experiments 1 and 2, except participants were instructed to select three (not two) traits per target face. The procedure for the augmented trials was identical to the baseline except for the addition of the AI’s recommendations, in the form of a brief natural language summary of the character of each person shown in the target images (examples are given in figure 1d).

#### 4.1.3. Materials

Compared with experiments 1 and 2, the following changes were made to let us introduce experimentally controllable and non-toxic forms of gender stereotyping into the output of GPT3.5 [67,68] (figure 1d). Methodologically, our aim in experiment 3 was to harness and gain experimental control over GPT3.5’s natural language capabilities, exploiting the set of trait attributes to simulate persuasive and stereotype-reinforcing or challenging bias patterns in a foundational LLM via prompt engineering. After training, LLMs need to be prompted, i.e. provided with an input stream of ‘tokens’ to produce an output [69]. In the case of GPT3.5, the output has an element of stochasticity due to its sampling from the distribution of possible and likely tokens that follow from the initial input [70]. Prompt engineering is the recently coined term for the process of systematically generating a sequence of prompts that seek to control and direct LLMs like GPT3.5 to perform particular tasks [71]. Without such prompting, an LLM is effectively inert, and prompt engineering is required in order to gain some control over the LLM, and in particular sufficient experimental control to rule out systematic confounds.

In experiment 3, we attempted to force GPT3.5 to include the character traits seeded via prompt engineering in their *verbatim* form, within brief gender-neutral character descriptions (see §4.1.4 for full details). Insisting, via the prompt engineering, that the seeded traits were used verbatim allows us a clear comparison with the results from experiments 1 and 2, to determine whether embedding the bias patterns in richer natural language distorts, mitigates or promotes the propagation of stereotype-reinforcing and challenging influences. However, by insisting on the verbatim use of the seeded character traits, we have thereby constrained GPT3.5’s ability to non-toxically express gender stereotypes or gender neutrality in natural language.

Therefore, in experiment 4, we removed the requirement to use the seeded traits verbatim, which then allowed GPT3.5 to complete the seeded prompts ‘as it wished’ (subject only to the same constraints on word count used in experiment 3—see §4.1.4). However, the seed traits themselves were retained within the prompts in experiment 4 because that is what allows us to experimentally control the patterns of bias that GPT3.5 expresses. Thus, experiment 4 lets us determine whether this technique can embed robust and persuasive bias patterns into a wider range of natural language, within the current ‘domain’ of character descriptions. It is worth signalling here that we can generalize this technique straightforwardly to a much wider range of domains, and that is the subject of current studies underway in our laboratory.

To allow GPT3.5 a richer basis for expression, we prompted it to create character descriptions using trait triples, not the trait pairs as were used in experiments 1 and 2. From a parent set of eight items, it is possible to draw 56 unique triples that provide us with exactly the correct number of triples needed for the experimental and practice trials. So we exploited that fact to produce all the unique three-trait triples from the top, male-linked eight, middle neutral eight and bottom female-linked eight traits based on their pre-rated GSA score. We then made 56 random combinations containing one triple of each type to form the required nine-trait response sets for each of the 28 baseline and 28 augmented trials (4 practice plus 24 experimental). This gave us, for each baseline and augmented trial, nine response traits, three of which were strongly female-linked, three of which were strongly male-linked and three of which were neutral. In the baseline trials, participants were instructed to select three traits reflecting their first impression of the individual shown in the accompanying facial image. Whereas in the following augmented phase, the system’s recommendations were the natural language descriptions that were produced by prompt engineering. As in experiments 1 and 2, these recommendations were shown at the beginning and throughout each augmented trial.

The facial images of young, White adults used in experiments 3 and 4 were again drawn from the CFD. Image selection was based on the database parameters ‘Prototypicality’ and ‘Unusualness’, for a prototypicality score above 3.5 (i.e. high end of prototypical) and unusualness score below 3 (i.e. low in unusualness), which gave us a pool of 99 facial images of different Caucasian individuals (52 men/47 women). To extract the required set of 28 male and 28 female images, the 99-image pool was first sorted by age (using the CFD age-data for each image), and the middle 56 images (28 men and 28 women) were then selected. We then scaled down the selected image sizes by a factor of 20% (resulting in images of 488 × 343 pixels) to allow room onscreen for the presentation of the natural language descriptions in augmented trials. As in experiments 1 and 2, the counterbalancing of traits to facial images was carried out by forming A and B subsets of the traits and the facial images, ensuring that they were used equally often in the baseline and augmented phases and that the nine-trait response sets were shown equally often with facial images of men and woman. This counterbalancing scheme is identical to that used in experiments 1 and 2 and so resulted in eight counterbalancing conditions. At the beginning of each online experiment session, each participant was randomly assigned by Testable to one of these eight conditions with equal probability.

As in experiments 1 and 2, the simulated bias conditions were implemented between-subjects, and we required three different conditions. One that contained a ‘fully reinforcing’ stereotypical bias, another that contained a ‘fully challenging’, counter-stereotypical bias and a third ‘neutral’ condition. The bias patterns were created by seeding GPT3.5 to produce character descriptions with strongly male- and female-linked traits taken from the top and bottom ends of the GSA scale, ensuring that facial images of men/women were always accompanied with recommendations for strongly male-/female-linked traits in the ‘fully reinforcing’ condition and vice versa for the ‘fully challenging’ condition. The neutral condition employed descriptions seeded by traits from the middle region of the GSA scale.

#### 4.1.4. Prompt engineering

The prompt engineering used Open AI’s application programming interface (API) to iteratively prompt GPT3.5 to generate brief character descriptions based on trait-triples passed to the model within the prompt. In all four experiments, our prompting used an emerging standard approach involving different sections for ‘Role’, ‘Task’, ‘Format’, ‘Example’ and ‘Data’. Role was specified as ‘You are a Face Recognition AI trained to describe people’s character traits’. Task was ‘Follow the instructions carefully. You will be shown pictures of faces and asked to provide character descriptions. You must not use gendered pronouns or reference any physical properties of the person. Lets begin the interaction. I am going to show you a picture of a face and you must try to describe the person’s character. However I will also give you three attributes to use in your verbatim description’. Format was ‘Keep the descriptions short to be about 30 words’. Example was ‘Here is an example: This person seems to be very committed to their goals and responsibilities. They also appear to be very cheerful and optimistic. Their unique and original personality shines through in their expression’. Data were ‘Here is the image of a face. The adjectives to use are <trait 1> <trait 2> <trait 3>’, with these final three variables populated by the various trait-triples.

The ‘role’ component specifies a face recognition AI trained to provide character traits using facial images, to force GPT3.5 to consider only the experimental stimuli available to participants, i.e. character traits and facial images. The ‘Task’ component focuses GPT3.5 on the individual character traits as seeds and constrains it to generate descriptions that rely on nothing other than facial images, excluding the use of gendered pronouns and reference to physical features. This is methodologically important for us because it allows us to map facial images of any men or women onto any character description without modification, removing the possibility that descriptions could be contradicted by facial characteristics not present or obvious. The ‘format’ component sets the word-length goal for descriptions that will fit onscreen in the experimental set-up. The ‘Example’ component then provides a sample description generated by GPT3.5 in initial prompt development work, which fits our criteria using neutral trait seeds. Finally, the end of the prompt specifies the data—the three seeds—that are to be used to describe a facial image.

Having constructed the prompt, an algorithmic process was then used in Python code to pass the trait-triples and check certain features of the character descriptions produced by the model until we had a set of descriptions using all the trait-triples that conformed to our requirements. The Python code iteratively prompted the model to produce a character description using each trait-triple and then checked the output against two key requirements. First, the number of words contained in the description had to be controlled to make sure the description was not too long to fit in the experiment’s screen layout and to make sure that the length of the descriptions in each bias condition was equated, on average. Algorithmic control of word count was needed due to GPT3.5’s inability to understand and control its own output length [72].

The prompt we used explicitly asks for descriptions of about 30 words in length, and the goal of the code was to iteratively prompt until the average length of male-typical, female-typical and neutral descriptions were approximately identical. Each description had to be based on three of the nine selectable traits shown on each augmented trial, and so in total there were 56 different, i.e. unique triples of each type (male-typical, female-typical and neutral). So we needed to generate 56 character descriptions of each type (168 in total), equating the average word count for each type so that the length of the AI’s recommendations was the same in every bias condition, on average.

We discovered via this process that GPT3.5 is not only poor at judging word count. The word count of the descriptions was also on average quite sensitive to whether triples were male-linked, female-linked or neutral in their semantics. For example, an apparent regularity we discovered was that descriptions with male-linked traits tended to be more verbose with longer word counts than the other two types. So for this reason, given our goal to produce equivalently long descriptions of each type, the number of iterations needed to converge to the goal of about 30 words on average was much higher for the male-linked than for the other two types. There may be some interesting underlying reason within GPT3.5 for this, possibly even an expression of gender bias in some form given it seems to be a difference between prompting with male- versus female-linked traits. However, we have not explored this particular ‘finding’ any further, although we could speculate that it may have some relation to GPT3.5’s still opaque (not in the public domain) training data and consequent understanding of male versus female stereotypes. An investigation of GPT3.5’s embedding space in this regard might be interesting [73].

The second key requirement for the character descriptions was how often they actually included the seed traits. In experiment 3, we sought to ensure that the seed traits were always used in the descriptions in their verbatim form. That is, in the grammatical form/tense of the seed trait itself. This was done to ensure that the recommendation actually contained three of the response options shown on each trial, as was the case in experiments 1 and 2. But we quickly discovered at the beginning of the prompt engineering that this simple requirement was actually beyond the capability of GPT3.5, even if the prompt explicitly asks for verbatim use of the seed traits. No matter how we tweaked the phrasing in the prompt, we could not be confident that the seed traits would always be used once we let the code iterate. We also discovered that the probability of verbatim use differed between the male-typical, female-typical and neutral descriptions, tending to show lower verbatim use in descriptions seeded with male-typical and neutral traits than female-typical traits. Our observation of apparent regularity might be an interesting pursuit for further work on GPT3.5’s embedding space, but given that our immediate purpose was to equate (i.e. remove) differences in verbatim use of the seeded traits for experiment 3, we modified the algorithm to approach as high a verbatim use as possible.

Via that process, our best result obtained mean verbatim use for the male-typical descriptions of 2.43 (s.d. = 0.50), for the female-typical descriptions 2.27 (s.d. = 0.80) and for the neutral 2.32 (s.d. = 0.54). Critically, independent-samples *t*-tests comparing these means were all non-significantly different (maximum *t*_110_ = 1.29, *p* = 0.2). It is worth pointing out that across all three types of description (male-typical, female-typical and neutral), we never got higher mean verbatim usage than 2.7 out of the three seed traits, and often much lower. This underscores the difficulty of constraining GPT3.5 to give us experimental control over the inclusion of the seed traits into the character descriptions, to transition from the base-trait recommendations used in experiments 1 and 2 to richer natural language recommendations using GPT3.5.

Quite clearly, we were restraining the expression of natural language deriving from the seed traits and therefore the expression of male and female stereotypes by GPT3.5. Therefore, to study the propagation of gender-stereotypical influence in a wider range of natural language, in the final experiment 4, we simply removed the restriction on verbatim use completely (while retaining the word count requirement) to produce ‘unrestrained’ but still seeded character descriptions. In terms of mean verbatim use (out of three) in experiment 4’s character descriptions, for the male-typical descriptions, it was 1.04 (s.d. = 0.95) for the female-typical descriptions 2.16 (s.d. = 0.97) and for the neutral 1.71 (s.d. = 0.89). Using independent-samples *t*-tests (two-tailed), compared with descriptions used in experiment 3, in experiment 4 there was significantly lower verbatim trait use in male-typical (*t*_110_ = 9.69, *p* < 0.001) and neutral (*t*_110_ = 4.36, *p* < 0.001) descriptions, but not for female-typical (*t*_110_ = 0.64, *p* = 0.52). Within experiment 4, female-typical verbatim use was significantly higher compared with neutral (*t*_110_ = 2.54, *p* = 0.01) and male-typical (*t*_110_ = 6.20, *p* < 0.001), while male-typical verbatim use was significantly lower than neutral (*t*_110_ = 3.90, *p* < 0.001).

### 4.2. Results

#### 4.2.1. Experiment 3

Participants were assigned randomly either to stereotype-reinforcing, challenging or neutral control AI-bias conditions. Aside from the use of brief character descriptions to explain the recommender’s decisions, the only change here versus experiments 1 and 2 was that participants were asked to select three (not two) response traits on each trial—reflecting our use of three seed traits to form each of ChatGPT’s character descriptions (§4.1.2).

**Figure 4.**
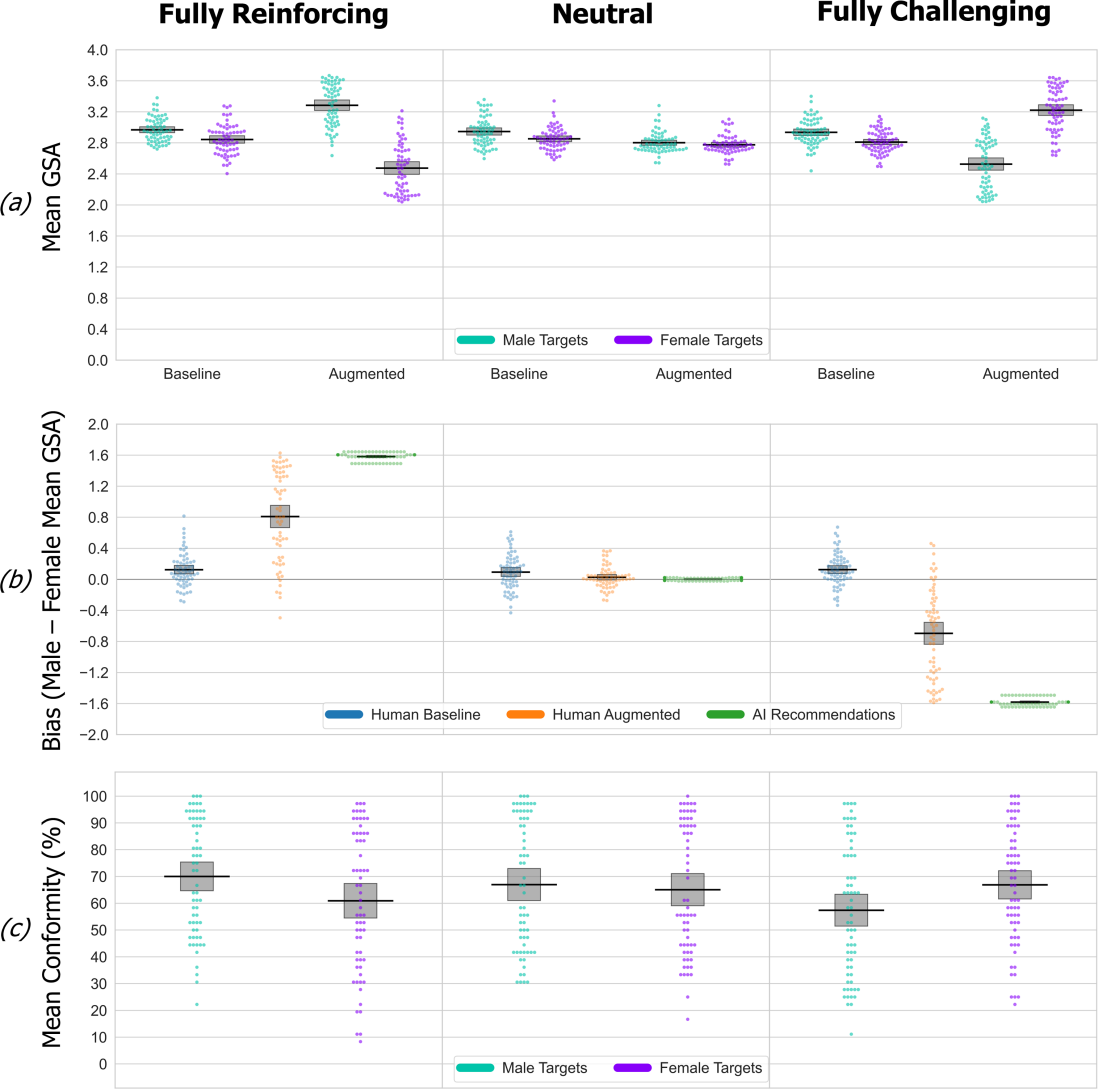
Experiment 3 data. See figure 2 legend for details. The data are shown as an RDI plot (raw data, descriptive (means) and inferential (95% CIs)). Here we use GPT3.5 to deliver stereotype-reinforcing, challenging and unbiased (stereotypically neutral) character descriptions in natural language. The findings fully replicate the bias amplifying and reversing effects found with the black-box (unexplained) recommendations in experiments 1 and 2. (*a*) Fully stereotype-reinforcing descriptions significantly increased the selection of stereotyped traits in the augmented phase, compared with baseline phase. Fully stereotype-challenging recommendations cause a reversal in participant’s bias compared with baseline. In the neutral control (unbiased) condition, descriptions reduced participant’s use of stereotypical male/female traits for male/female targets. Consequently, the augmented phase mean GSA scores for male and female targets now converge. (*b*) Participants have a consistent, significant, baseline bias in each simulated AI bias condition, which qualitatively changes in each augmented phase under the influence of GPT3.5’s recommendations. (*c*) The different simulated AI-bias patterns in GPT3.5 had no effect on conformity to its recommendations about female targets, whereas conformity to its recommendations about male targets was significantly higher in the fully reinforcing versus challenging condition.

Experiment 3 findings are summarized in figure 4. We first carried out an initial mixed-design ANOVA on the mean GSA scores from the baseline and augmented phases (figure 4*a*), using between-group factors of simulated AI-bias and within-participant factors of target gender (male versus female) and phase (baseline versus augmented). There was a significant three-way interaction (*F*_2,189_ = 138.55, *p* < 0.001, partial *η*² = 0.60), producing an effect size comparable to that in experiment 2.

First, once again we find that baseline bias (i.e. mean male–female GSA score at baseline) is statistically significant in all three groups (all *p*s < 0.001, Bonferroni-corrected) and does not differ between groups (*t*s_124_ ≤ |0.77|, *p*s ≥ 0.44, Cohen’s *d* ≤ 0.14). Second, the mean GSA scores in the augmented phase all significantly differed from their corresponding baseline values in all three simulated AI-bias conditions. When GPT3.5’s character descriptions reinforce gender stereotypes, this significantly amplifies (all *p*s < 0.001, Bonferroni-corrected) participant’s baseline bias (e.g. compare left panel in figures 3*a* and 4*a*), precisely as observed in experiments 1 and 2. Significant reversal of the baseline pattern was also observed when GPT3.5 described people’s character in ways that directly challenge gender stereotypes (all *p*s < 0.001, Bonferroni-corrected), again replicating the prior findings (e.g. compare right panel in figures 3*a* and 4*a*). Seeding GPT3.5 with gender-neutral character traits (not strongly associated with either male or female stereotypes) as a control also significantly changed participant’s augmented phase mean GSA scores versus baseline (*p*s ≤ 0.033, Bonferroni-corrected), effectively eliminating participant’s baseline bias completely (figure 4*a*, middle). It’s worth emphasizing that this outcome is highly desirable and valuable even in isolation from our other findings. Demonstrating that a neutralizing effect on pre-existing human bias can be captured experimentally opens a new route to explore factors that aid or hinder the goal of producing persuasive, debiased generative AI.

**Figure 5.**
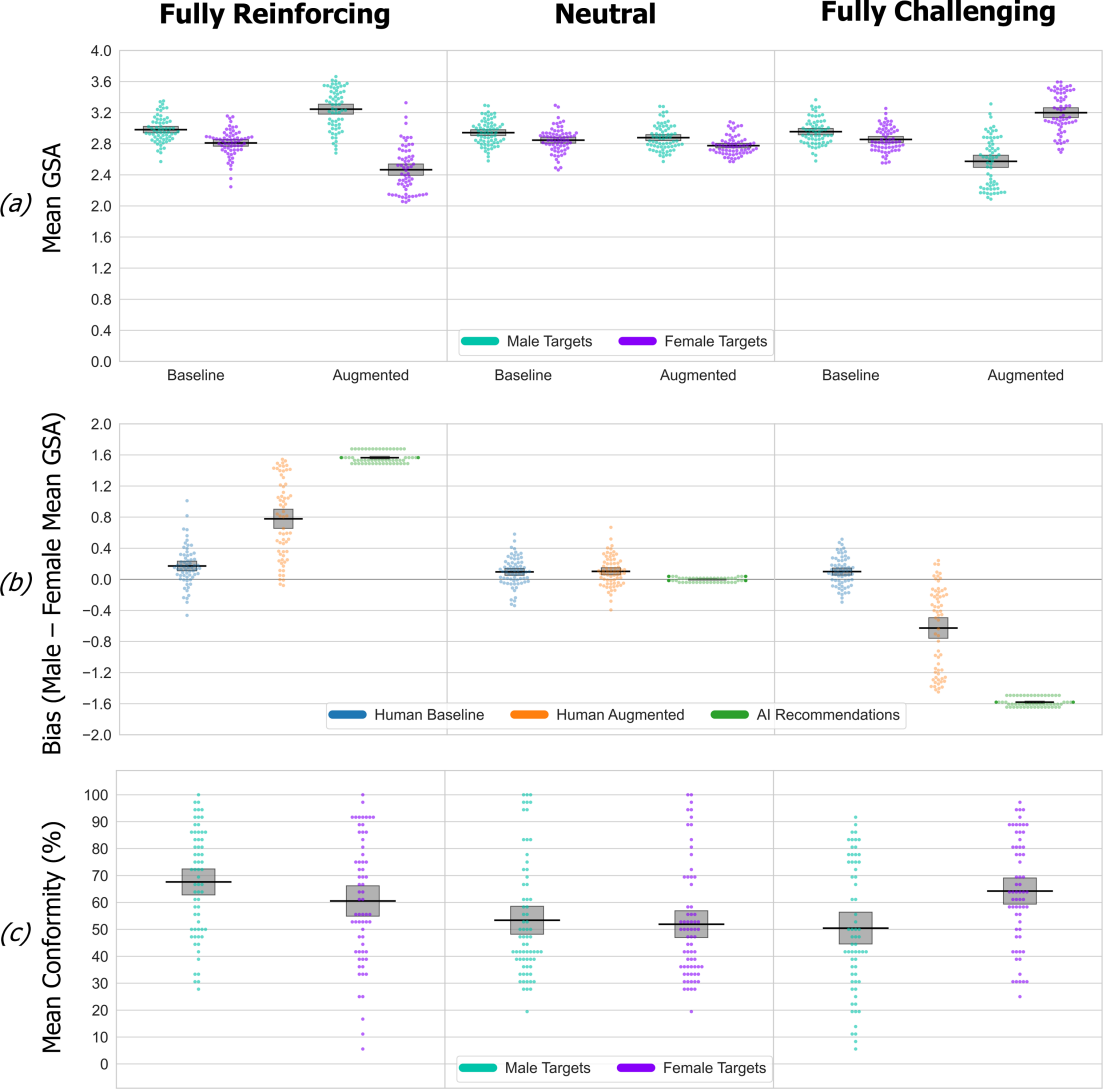
Experiment 4 data. See figure 2 legend for details. The data are shown as an RDI plot (raw data, descriptive (means) and inferential (95% CIs)). Relaxing the prompting constraints on how GPT3.5 expresses seeded male and female character stereotypes. The findings, with one exception, entirely and consistently replicate the patterns observed in experiment 3 when GPT3.5 was constrained to use the seed traits verbatim. Namely, neutral descriptions did not eliminate participants baseline bias, which is still present and quite intact in the augmented phase.

Figure 4*b* summarizes the qualitatively and significantly different (*t*s_127_ ≥ |9.54|, *p*s ≤ 0.001, Cohen’s *d* ≥ 1.68) augmented phase bias induced in participants in each of the simulated AI bias conditions, highlighting the complete absence of detectable bias in the augmented phase of the unbiased control condition (figure 4*b*, middle; *t*_62_ = 1.49, *p* = 0.14 (two-tailed), against null value = 0, Cohen’s *d* = 0.19). Propagation of the simulated AI’s bias into participant’s augmented phase decisions, as a proportion of the total available bias in the AI’s recommendations, is reduced in the stereotype-challenging compared with reinforcing condition (reinforcing = 0.51 (s.d. = 0.37) versus challenging = 0.45 (s.d. = 0.37)) mirroring the significant reduction observed in experiments 1 and 2. Here, however, the reduction is not statistically significant (*t*_127_ = 1.14, *p* = 0.26, Cohen’s *d* = 0.2).

GPT3.5, apparently, is more persuasively conveying a stereotype-challenging influence than was achieved by the black-box recommendations in experiments 1 and 2. We can directly test this interpretation by comparing the propagation of stereotype-challenging influence here versus in experiment 2. This across-study comparison confirmed that GPT3.5 is significantly better at propagating the stereotype-challenging influence than unexplained black-box recommendations (*t*_159_ = 2.87, *p* = 0.005, Cohen’s *d* = 0.46), but not at propagating the stereotype-reinforcing influence (*t*_157_ = 0.84, *p* = 0.40, Cohen’s *d* = 0.14). Stereotype-challenging advice, therefore, specifically benefits in persuasive power from natural language explanation by GPT3.5.

We replicate the findings from experiments 1 and 2 of reduced conformity to male but not female stereotype-challenging versus reinforcing recommendations (figure 4*c*). Analysed using ANOVA, the conformity rates produced an interaction between target gender and simulated AI-bias condition (*F*_2,189_ = 37.05, *p* < 0.001, partial *η*² = 0.28). Here, once again, simulated AI-bias pattern has no effect on conformity to recommendations about female targets (*p*s ≥ 0.49, Bonferroni-corrected), whereas conformity to recommendations about male targets is significantly higher in the fully reinforcing versus challenging condition (*p* = 0.008, Bonferroni-corrected).

Comparison of GPT3.5’s persuasiveness versus that of recommendations in experiment 2 revealed that GPT3.5’s stereotype-challenging recommendations about male targets attracted significantly higher conformity than their black-box (unexplained) counterparts (*t*_159_ = 2.26, *p* = 0.026, Cohen’s *d* = 0.36). The same is true for GPT3.5’s enhanced ability to transmit a stereotype-challenging message for female targets versus black-box recommendations in experiment 2 (*t*_159_ = 2.72, *p* = 0.007, Cohen’s *d* = 0.44). But when reinforcing stereotypes, there is no difference in the rate of conformity to GPT3.5 explanations versus black-box recommendations (male stereotype reinforcing, *t*_157_ = 1.51, *p* = 0.13, Cohen’s *d* = 0.24; female stereotype reinforcing, *t*_157_ = 0.29, *p* = 0.78, Cohen’s *d* = 0.05).

Overall, we find that GPT3.5 is a more effective propagator of stereotype-challenging content about men and women than black-box recommendations. It is important to point out that this enhanced persuasiveness is not caused merely by us seeding GPT3.5 with trait triples versus the trait pair recommendations used in experiments 1 and 2. If the use of triples as seeds had a more powerful persuasive effect *per se*, we would have seen the same enhanced persuasiveness pattern for GPT3.5’s stereotype-reinforcing influence, but we did not.

#### 4.2.2. Experiment 4

In experiment 4, we again use prompt engineering to seed GPT3.5 with the character traits in order to systematically control the direction of its gender bias, but we remove the constraint to use the seed traits verbatim (see §4.1.4 for full details). In all other respects, experiment 4 was identical to experiment 3. The findings are summarized in figure 5. An initial mixed-design ANOVA was carried out on the GSA scores (figure 5*a*) using between-group factors of simulated AI-bias condition and within-participant factors of target gender (male versus female) and phase (baseline versus augmented). We found a significant three-way interaction (*F*_2,193_ = 131.2, *p* < 0.001, partial *η*² = 0.58) with effect size comparable to experiment 3.

Within each AI-bias condition, baseline bias (male–female mean GSA score) is statistically significant (maximum *p* = 0.002, Bonferroni-corrected). Baseline bias in the reinforcing condition (figure 5*a*, left) trends higher than in the neutral (figure 5*a*, middle; *t*_130_ = 2.0, *p* = 0.051 (two-tailed), Cohen’s *d* = 0.34) and challenging (figure 5*a*, right) conditions (*t*_126_ = 1.82, *p* = 0.08 (two-tailed), Cohen’s *d* = 0.32) but did not differ between neutral and challenging (*t*_130_ = 0.12, *p* = 0.90, Cohen’s *d* = 0.02). The stereotype-reinforcing recommendations significantly increased both the male and female mean GSA scores in the augmented phase compared with their corresponding baseline scores (all *p*s < 0.001, Bonferroni-corrected), hence amplifying participant’s baseline bias. Meanwhile, stereotype-challenging recommendations significantly reverse the mean male and female GSA scores in the augmented baseline phases (all *p*s < 0.001, Bonferroni-corrected). In the neutral condition, baseline bias is no longer effectively neutralized by recommendations. In fact, the augmented phase bias (male–female mean GSA score; (figure 5*a*, middle) is still significant in the neutral condition (*p* = 0.042, Bonferroni-corrected). These shifts in bias pattern induced in each augmented phase (figure 5*b*) are all significantly different from each other (*t*s_130_ ≥ |10.31|, *p*s ≤ 0.001, Cohen’s s *d* ≥ 1.8). Propagation of the stereotype-reinforcing simulated bias pattern into participant’s augmented phase decisions is again higher than the stereotype-challenging influence (0.51 (s.d. = 0.32) versus 0.40 (s.d. = 0.34)), but the reduction is a much weaker effect (*t*_126_ = 1.71, *p* = 0.09, two-tailed, Cohen’s *d* = 0.3) than observed in experiments 1 and 2. Directly comparing the propagation of stereotype-reinforcing and challenging influences here versus in experiment 3, no statistically detectable differences were observed (*t*s_128_ ≤ |0.68|, *p*s ≥ 0.5, Cohen’s *d* ≤ 0.12). That is, GPT3.5 continues to be more persuasive in challenging stereotypes, compared with the black-box recommendations in experiments 1 and 2.

In the conformity data (figure 5*c*), we replicate the now familiar pattern of reduced conformity to male but not female stereotype-challenging versus reinforcing recommendations. Analysis of the conformity rates via ANOVA produced a target gender and simulated AI-bias interaction (*F*_2,193_ = 34.37, *p* < 0.001, partial *η*² = 0.26). Conformity to reinforcing or challenging recommendations does not differ for female targets (*p* = 0.99, Bonferroni-corrected), whereas conformity to recommendations about male targets is significantly higher in the fully reinforcing versus challenging condition (*p* < 0.001, Bonferroni-corrected). For the unbiased neutral recommendations, these are less persuasive than challenging recommendations for female targets (*p* < 0.003, Bonferroni-corrected) and reinforcing recommendations for males (*p* < 0.001, Bonferroni-corrected). In the unbiased neutral condition, GPT3.5’s descriptions here attracted significantly lower conformity both for male and female targets compared with the verbatim use of neutral seed traits in experiment 3 (*t*s_129_ ≥ |3.33|, *p*s ≤ 0.001, Cohen’s *d* ≥ 0.58).

### 4.3. Discussion (experiments 3 and 4)

The key outcome from experiment 3 is that our prompt engineering of GPT3.5 resulted in significant amplification, neutralization and reversal of human baseline stereotypical bias (summarized in figure 4*b*). Our approach therefore allows the controlled manipulation of non-toxic and highly persuasive patterns of gender-stereotypical bias, neutrality or counter-stereotypical suggestions into GPT3.5’s rich natural language output. These findings directly confirm societal concerns about the impact of human interaction with stereotypically biased generative AI and go beyond recent demonstrations of GPT3.5’s propensity for non-toxic stereotypical bias [18–21,23,24] by clearly showing that in the absence of mitigation or further safeguards, GPT3.5 output significantly amplifies baseline human stereotypical bias.

Alongside that concerning conclusion, two other pieces of evidence emerge from experiment 3 to converge on a second, somewhat more positive conclusion. Stereotype-challenging messages are significantly more effective when conveyed by GPT3.5, even against the apparently more robust male stereotype, in comparison with black-box recommendations. Similarly, GPT3.5’s unbiased neutral character descriptions successfully nullified human baseline bias within the task, eliminating it entirely from augmented decisions. These findings show that counter-stereotypical messaging can be conveyed by generative AI just as persuasively as stereotypical messaging and that gender bias can be eliminated entirely, in groups of human participants with significant, pre-existing stereotypical bias. Experimentally at least, it is possible to overcome pre-existing human stereotypical bias and the reluctance that it induces to accept neutral or counter-stereotypical messaging, when such messaging is conveyed in a near-verbatim form of the character traits. Of course, our experimental control raises the question of what happens when GPT3.5 can more freely express male and female character stereotypes, without any constraint on the verbatim use of the seed traits.

The answer was revealed in experiment 4, where we allowed GPT3.5 freedom to express male and female character stereotypes, albeit still seeded by character traits. We observed essentially the same patterns of bias amplification and reversal, without any weakening or distortion compared with the patterns observed in experiment 3 (summarized in figure 5*b*). Also, as in experiment 3, GPT3.5’s less constrained prompting conveyed stereotype-challenging influences just as effectively as stereotype-reinforcing influences. All in all, experiment 4 replicates experiment 3 in every respect, except that the recommendation seeded with neutral traits did not neutralize participant’s baseline bias during the augmented phase. The reason is that participants selected (i.e. conformed to) unbiased neutral traits from among the available response options significantly less often than they did in experiment 3. This finding exposes a sensitivity to initial prompt conditions specific to GPT3.5’s elaboration upon the neutral traits that are particularly important, assuming that one’s aim is to produce a *persuasive* model or application to counteract or neutralize the existing influence of gender stereotypes within human decision-making.

## 5. General discussion

Our aim has been to contribute constructively towards the safe development of fair AI by experimentally testing the actual influence of simulated AI bias patterns within human–AI interaction relative to a known level of baseline human bias. Although this experimental approach entails abstraction away from consequential real-life decision-making, that sacrifice provides explicit control over conditions that could elicit hypothetical and concerning phenomena, like amplification of human bias by similarly biased AI systems. This has allowed us to provide, for the first time, experimental confirmation of bias amplification, replicated multiple times in different, diverse adult samples, albeit English-speaking and focused on stereotypes applied to images of White men and women.

Our approach can now readily extend in a variety of ways to explore stereotypical influences on other tasks and types of human–AI interaction and in sub-groups with different baseline bias patterns or with additional dependent measures of interest. One particularly timely direction for future work is to examine generative AI’s amplification of bias deriving from other singular stereotypes, e.g. age, race or disability, and their intersectional biases [58,59,69]. The need for this is urgent, driven by accumulating evidence of corresponding stereotypical biases in the output of advanced generative AI models like GPT3.5 [18–24]. Our current findings strongly suggest that interacting with such biased systems will amplify baseline bias levels and that specific prompting regimes should be able to successfully reverse or neutralize baseline bias. A further extension of the approach will be to examine bias propagation in more extended conversations supported by LLMs, going beyond the strictly controlled prompting approach used here, and such work is currently underway in our laboratory.

By empirically demonstrating the authenticity of bias amplification by generative AI, we provide direct support for significant public and expert concerns about the societal impact of biased AI. Our experimental approach also offers some basis for balance in debates focused on the negative impact of biased AI, by showing that human stereotypical bias can be neutralized—and even reversed—within human–AI interaction. This provides novel motivation and support for continued efforts to apply bias mitigation or debiasing techniques wherever possible to generative AI models. Indeed, it seems reasonable and empirically defensible now to assume that systems expressing gendered stereotypes will amplify baseline human bias in the absence of debiasing or other mitigation efforts. In fact, given our findings, the release of a system without such robust safeguards in place, and some means of assessing baseline bias, requires clear justification. For example, the release has benefits that somehow outweigh the consequences of bias amplification. Further empirical evidence, for example, concerning propagation of other forms of stereotypical bias, should in due course strengthen and extend the empirical basis for such improvements in the regulation of human–AI interaction [15,60,61].

A limitation of our current experiments is that they do not address the underlying psychological mechanisms that convey, moderate or enhance stereotype-reinforcing or challenging influences. This is addressed in our recent prior work [61], using a psychological distinction established in human–human interaction between ‘normative’ versus ‘informational’ conforming influences [32,36,37,39,61]. Normative influences stem from our desire to maintain social bonds and cooperative aid with like-minded individuals, which has deep, adaptive biological roots [32,33,39]. Conformity under normative influence signals our adherence to social conventions and could well contribute to the propagation of AI’s stereotypical influence.

Informational influences from a system during human–AI interaction stem from the need to maintain an accurate mental model of the world to support adaptive behaviour [32,33]. Informational influence should not be conflated with the accuracy of an AI system (or any other information source). We know it is futile to conflate informational influence with the objective accuracy of an information source from many years of work examining conformity in human–human interaction, where sources lacking in objective accuracy are misperceived as accurate and are powerfully persuasive [35–37,40–43]. A system’s informational influence is therefore not at all likely to be a property of its accuracy alone and instead reflects a trade-off that balances our own knowledge and credibility against that of the AI system, as we have shown in human–human interaction [41] and recently in human–AI interaction [61]. Stereotypical bias amplification, neutralization and reversal via informational influences are therefore critical psychological mechanisms to explore in future work.

In conclusion, we would like to return to a point raised in §1 about technocentric solutions to the problem of biased AI, in the form of refined model architectures or cleansing of training data. In our approach here, we adopt an alternative kind of reasonably advanced technical method, i.e. experimental psychology, to demonstrate human susceptibility to gender stereotypical bias that we experimentally controlled in the natural language output by a leading LLM, GPT3.5. This, we believe, now lends clear and strong empirical evidence to calls for regulation and monitoring of AI deployments where stereotypes, particularly of traditional binary gender roles, may already influence human decision-making. Our findings clearly show that such biases in recommending AI systems can significantly amplify existing gender stereotypical influences. Our findings also clearly show that human baseline bias can be neutralized or even reversed, if a recommending system is ‘debiased’ or challenges such stereotypes, although the persuasive effect is reduced for male compared with female stereotypes. This does help to support continued efforts to mitigate such biases through technocentric approaches in machine learning because our data show that these are likely to be effective in counteracting human baseline bias. However, none of this addresses the systematic structures of marginalization, perhaps supported by the cognitive shortcuts that we call stereotypes, in society itself. But, by directly investigating and demonstrating bias amplification within human–AI interaction, we hope that our findings will help to focus attention on the very real possibility that biased AI will trigger vicious cycles that make existing societal prejudices and discrimination worse.

## Data Availability

Our experimental data and materials can be accessed via the OSF [65].

## Footnotes

^1^
Due to a coding error, 32 participants could not select an age below 64 from the dropbox provided during the initial demographic questionnaire, but for 30 of these participants, we were able to estimate their age from the year of birth information they provided at the end of the experiment.
